# Drug-induced cerebral glucose metabolism resembling Alzheimer’s Disease: a case study

**DOI:** 10.1186/s12888-015-0531-9

**Published:** 2015-07-11

**Authors:** Matthias W. Riepe, Britta Walther, Catharina Vonend, Ambros J. Beer

**Affiliations:** Division of Mental Health & Old Age Psychiatry, Psychiatry II, University of Ulm, Ludwig-Heilmeyer-Strasse 2, D-89312 Günzburg, Germany; Department of Nuclear Medicine, Ulm University, Albert-Einstein Allee 23, D-89081 Ulm, Germany

**Keywords:** Alzheimer’s disease, Recurrent depressive episode, Lithium, Primidone, FDG-PET, Amyloid PET

## Abstract

**Background:**

With aging of society the absolute number and the proportion of patients with cognitive deficits increase. Multiple disorders and diseases can foster cognitive impairment, e.g., Alzheimer’s disease (AD), depressive disorder, or polypharmacy.

**Case presentation:**

A 74 year old man presented to the Old Age Psychiatry Service with cognitive deficits while being treated for recurrent depressive episodes and essential tremor with Venlafaxine, Lithium, and Primidone. Neuropsychological testing revealed a medio-temporal pattern of deficits with pronounced impairment of episodic memory, particularly delayed recall. Likewise, cognitive flexibility, semantic fluency, and attention were impaired. Positron emission tomography (PET) with fluorodeoxyglucose was performed and revealed a pattern of glucose utilization deficit resembling AD. On cessation of treatment with Lithium and Primidone, cognitive performance improved, particularly episodic memory performance and cognitive flexibility. Likewise, glucose metabolism normalized. Despite normalization of both, clinical symptoms and glucose utilization, the patient remained worried about possible underlying Alzheimer’s disease pathology. To rule this out, an amyloid-PET was performed. No cortical amyloid was observed.

**Conclusion:**

Pharmacological treatment of older subjects may mimic glucose metabolism and clinical symptoms of Alzheimer’s disease. In the present case both, imaging and clinical findings, reversed to normal on change of treatment. Amyloid PET is a helpful tool to additionally rule out underlying Alzheimer’s disease in situations of clinical doubt even if clinical or other imaging findings are suggestive of Alzheimer’s disease.

## Background

Alzheimer’s disease (AD) is a common cause for cognitive impairment in old age [1]. Cognitive impairment may also result from prescription of inappropriate drugs in the elderly [2–4]. Long-term prescription of anticholinergic drugs increases the risk for developing dementia and AD [5, 6]. Thus, in elderly patients both needs to be considered as a differential diagnosis of AD, cognitive impairment as an acute or subacute side effect of inappropriate medications, and dementia or AD on long-term treatment with inappropriate medications.

At onset of AD, medio-temporal functions are reduced [7, 8]. With spread of disease other functions are also affected and a stage-dependent pattern of deficits unfolds [9]. In terms of function, episodic memory, in particular delayed recall, is compromised first, followed by impairment of executive functions and visuo-construction [9–11]. Once a cognitive deficit commences, the natural course of the disease goes along with an increase of severity over time without major fluctuation of severity and without reversal of symptoms [9–11].

Several neurochemical and imaging biomarkers are established to capture either amyloid metabolism or neurodegeneration in AD [12]. Structural imaging targets visualization of neurodegeneration. While no structural imaging feature has perfect sensitivity or specificity, diagnostic algorithms using structural imaging have been proposed to support the diagnosis of AD and differential diagnosis of other dementias [13]. Likewise, imaging of glucose metabolism or amyloid deposition can contribute to the diagnosis and differential diagnosis of dementia disorders [14]. Imaging with amyloid tracers has provided a means to directly visualize amyloid in the brain and has been found well enough established to be used in criteria recommended for diagnosing AD [15, 16]. At present, however, it remains under discussion whether different amyloid ligands perform equivalently [17, 18].

Decreased temporo-parietal glucose utilization as measured with positron emission tomography with fluorodeoxyglucose (FDG-PET) is considered specific for Alzheimer’s Disease [19, 20]. The degree of glucose hypometabolism correlates with performance in appropriate neuropsychological tests [21].

Lithium is a widely used drug recommended for treatment of bipolar disorder even in the elderly [22]. Its clinical side effects, however, remain ambiguous. While some reports demonstrate an impaired memory on treatment with Lithium [23–25], another report in a small number of patients finds memory unaffected on treatment with Lithium [26]. Likewise, the findings on the effect of Lithium on cerebral glucose metabolism are ambiguous. An increased glucose metabolism was reported in rodents [27] while another study reported glucose utilization to be unchanged on low dosages of Lithium and inhibited on higher dosages of Lithium [28]. In humans, the evidence on glucose utilization is sparse - a decrease in the hippocampus and the cerebellum was reported [29] but it remained unclear, whether reduction in glucose metabolism was related to clinical symptoms [29]. In contrast, an increase of glucose utilization on treatment with Lithium was found in the posterior cingulate and in the orbitofrontal and dorsolateral frontal cortex in pathological gamblers [30]. On intoxication with Lithium, a reduction of cerebral glucose metabolism resembling Alzheimer’s disease has been found [31].

Primidone is a standard medication for treatment of essential tremor [32]. While cognitive side effects of treatment with Primidone are frequent in the elderly [33], the exact pattern of cognitive impairment on treatment with Primidone has not been characterized. In humans, an increase of glucose utilization was found on cessation of treatment with Primidone [34]. However, the increase of glucose utilization did not show a specific pattern but rather was observed in all but one area of the brain.

Here we report a patient that showed both, a pattern of cognitive deficits and a pattern of glucose utilization resembling Alzheimer’s disease while being treated for recurrent depressive episodes and essential tremor with Venlafaxine, Primidone, and Lithium.

## Case presentation

A 74 year old man presented to the Old Age Psychiatry Service with cognitive deficits, in particular deficits of short term memory, while being treated for recurrent depressive episode. Depressive disorder was diagnosed 1986 and under continuing treatment with Moclobemid the depression was remitted until 2009. Several month after cessation of treatment with Moclobemid depression recurred and he was treated with Valproic acid and Sertraline. Due to incomplete remission, several antidepressants were used until 2014. On admission the patient was treated with Lithium (serum level 0.8 mmol/l (norm: 0.6 – 1.0 mmol/l)) and Venlafaxine. In addition, the patient had familial essential tremor treated with Primidone.

Neuropsychological testing on admission (Table 1) revealed normal performance on cognitive (Mini-Mental Status Examination 28/30) and affective scales (Geriatric Depression Scale 3/15). Digit and block span forward and backward were 8 and 5, and 6 and 5, respectively. The German version of the California Verbal Learning Test showed impaired word learning (3/16) in trial 1 with impaired delayed recall (4/16). Trail-Making Test A and B were 121 and 278 s, respectively. Semantic fluency was reduced to 13 animals per minute.

**Table 1:** Neuropsychological findings at baseline and after cessation of treatment with Lithium and Primidone

	Baseline	Follow-up
MMSE	28	30
Digit span forward	8	9
Digit span backward	5	6
Block span forward	6	7
Block span backward	5	7
Trail-Making Test A	121	78
Trail-Making Test B	278	103
CVLT I	3	4
CVLT V	4	9
Delayed recall	3	8
Semantic fluency (animals)	13	21
Phonematic fluency (P)	6	13
Phonematic fluency (S)	11	20

After cessation of treatment with Lithium and Primidone, the impairment on neuropsychological testing reversed (Table 1). Digit and block span forward and backward improved to 9 and 6, and 7 and 7, respectively. Verbal learning improved to 4 of 16 in trial I and 9 of 16 in delayed recall. Trail-Making Test A and B were 78 s and 103 s, respectively. Semantic fluency improved to 23 animals per minute.

**Fig. 1:**
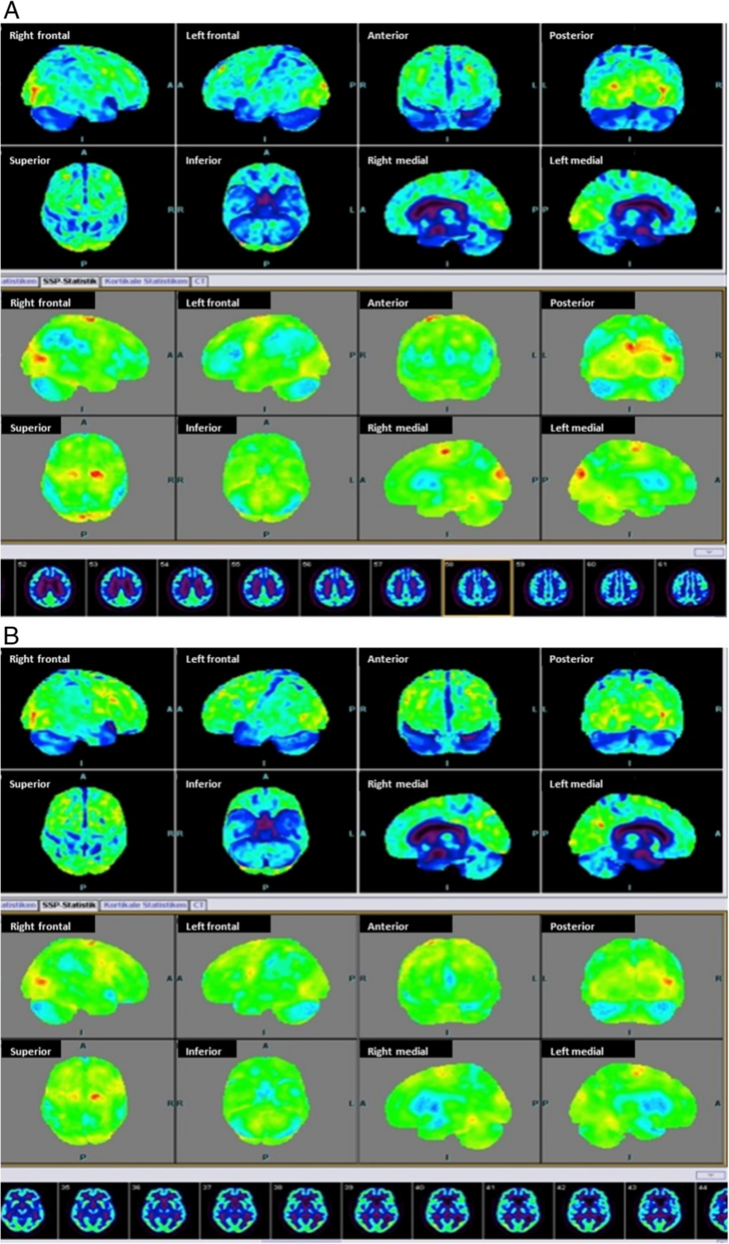
FDG-PET on **a** May,23rd, with an Alzheimer like glucose utilization deficit in temporo-parietal regions and the posterior cingulum and **b** June, 25th, with a normalization of glucose utilization in all brain regions

FDG-PET on admission showed reduced glucose metabolism in temporo-parietal areas and the posterior cingulum with a pattern characteristic for Alzheimer’s disease. After cessation of treatment with Lithium and Primidone glucose utilization in FDG-PET normalized in all areas (Fig. 1).

**Fig. 2:**
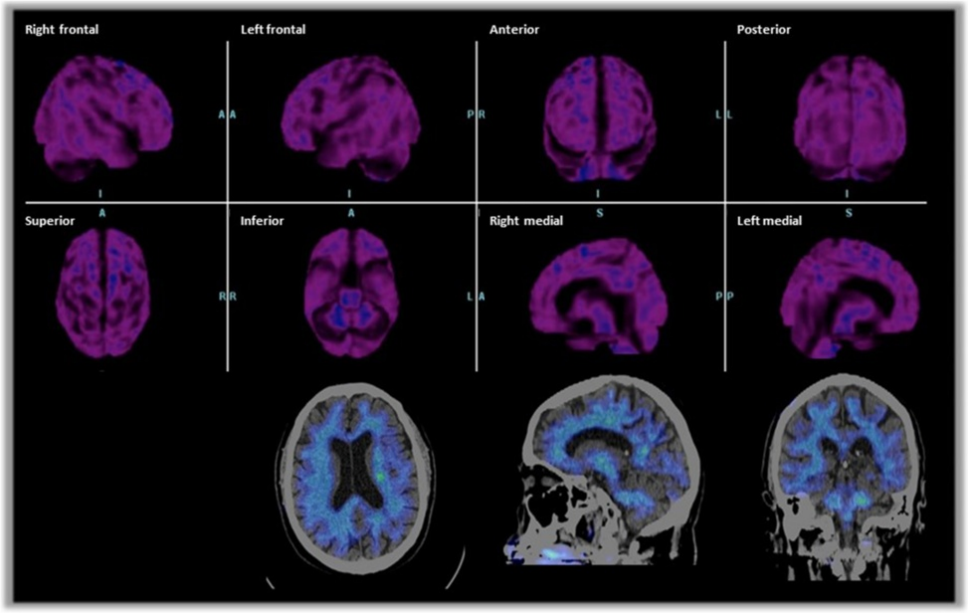
Amyloid-PET on June, 25th, demonstrating the absence of cortical amyloid in all brain regions

To further rule out Alzheimer pathology an amyloid-PET with was performed. No cortical amyloid deposition was observed and thus, AD was ruled out (Fig. 2).

## Discussion

Due to demographic changes an ever rising number of elderly patients present to Medical Services with cognitive impairment. Both, neurodegenerative diseases, amongst which AD is the most common aetiology of dementia in old age, and inappropriate medications [4] are frequent causes of cognitive impairment in old age. Several lists have been proposed to check for inappropriate medications in the elderly ever since the Beers criteria [35] have been brought forward [36–39].

The diagnostic algorithm in the case presented here cannot be generalized. Considering the known impact of potentially inadequate drugs on cognition, it is in general a straightforward procedure to cessate treatment with these drugs or reduce their dosage. Due to changes in pharmacokinetics and pharmacodynamics it may be worthwhile in general to consider decrease of dosage in the elderly even when serum concentration of suspicious drugs are within the therapeutic range [40, 41], but no clear-cut recommendations are established. In the present case, however, the patient had had multiple complications on change of prior medications: on change of antidepressive treatment several years ago the clinical situation of the patient had worsened and at first the patient was reluctant to change antidepressive medication again. Likewise, essential tremor was burdensome for the patient and while treatment with propranolol was tried some years ago it had to be abolished for reasons of cardiac side effects so that the patient was reluctant to change treatment with Primidone.

Both, neuropsychological assessment and positron emission tomography imaging, have characteristic patterns in the early stages of Alzheimer’s disease. Cognition at onset of disease is characterized by a medio-temporal deficit [9] with predominant deficits of episodic memory (see above). At this stage, FDG-PET is characterized by a temporo-parietal glucose hypometabolism [19, 20]. Both findings were present in the patient described above and both findings reverted to normal when treatment with Primidone and Lithium was stopped.

It has been reported that Primidone reduces cerebral glucose metabolism [34]. However, reduction of glucose metabolization under treatment with primidone affects almost all brain regions and cannot explain a specific pattern of glucose utilization deficit such as the one observed in the patient reported here. In contrast, a pattern of glucose utilization deficit resembling Alzheimer’s disease has been reported on intoxication with Lithium [31]. However, Lithium levels in the patient reported here were within the therapeutic range as were serum levels of Primidone. Thus, this case is the first to demonstrate that an Alzheimer-like pattern of glucose utilization deficit can occur while psychotropic and neurotropic substances are within normal range.

The course of clinical symptoms and glucose utilization findings on cessation of treatment with Lithium and Primidone argued that the Alzheimer-concordant neuropsychological and imaging findings were a false positive finding due to the pharmacological treatment. We substantiated this interpretation with amyloid PET where no cortical amyloid whatsoever was observed.

Half a year after the diagnostic procedures and change of therapy the patient reported to be in good mood and to have no impairment in everyday functions, in particular no impairment with eating and writing, due to residual symptoms of essential tremor.

## Conclusion

Pharmacological treatment of older subjects may mimic glucose metabolism and clinical symptoms resembling Alzheimer’s disease – this further substantiates the general recommendation to reduce or cessate treatment with potentially harmful drugs known to impair cognition in the elderly. Both, imaging and clinical findings reversed on cessation of treatment. Amyloid PET is a helpful tool to additionally rule out underlying Alzheimer’s disease in situations of clinical doubt even if clinical or other imaging findings are suggestive of Alzheimer’s disease.

## Consent

Written informed consent was obtained from the patient for publication of this case report and any accompanying images. A copy of the written consent is available for review by the Series Editor of this journal.
